# High Performance Polymer Solar Cells Using Grating Nanostructure and Plasmonic Nanoparticles

**DOI:** 10.3390/polym14050862

**Published:** 2022-02-22

**Authors:** Ali Elrashidi, Khaled Elleithy

**Affiliations:** 1 Department of Electrical Engineering, University of Business and Technology, Jeddah 21432, Saudi Arabia; 2 Department of Engineering Physics, Alexandria University, Alexandria 21544, Egypt; 3 Department of Computer Science and Engineering, University of Bridgeport, 221 University Ave., Bridgeport, CT 06604, USA; elleithy@bridgeport.edu

**Keywords:** FDTD, plasmonic nanoparticles, polymer solar cell, short circuit current density, power conversion efficiency

## Abstract

This work introduces a high-efficiency organic solar cell with grating nanostructure in both hole and electron transport layers and plasmonic gold nanoparticles (Au NPs) distributed on the zinc oxide (ZnO) layer. The periods of the grating structure in both hole and electro transport layers were optimized using Lumerical finite difference time domain (FDTD) solution software. The optimum AuNP radius distributed on the ZnO layer was also simulated and analyzed before studying the effect of changing the temperature on the solar cell performance, fill factor, and power conversion efficiency. In addition, optical and electrical models were used to calculate the short circuit current density, fill factor, and overall efficiency of the produced polymer solar cell nanostructure. The maximum obtained short circuit current density and efficiency of the solar cell were 18.11 mA/cm^2^ and 9.46%, respectively, which gives a high light absorption in the visible region. Furthermore, the effect of light polarization for incident light angles from *θ* = 0° to 70° with step angle 10° on the electrical and optical parameters were also studied. Finally, optical power, electric field, and magnetic field distribution inside the nanostructure are also illustrated.

## 1. Introduction

Organic photovoltaic solar cells have drawn much research interest in the last years due to their flexibility, low cost, lightweight, and production compatibility [1,2,3]. However, the organic solar cell stability and its lower efficiency are considered as the main challenges [4]. As a result, a lot of research has been conducted to improve the solar cell performance, especially the power conversion efficiency (PCE), by plasmonic cavity [5], optimizing the device structure [6] and using graphene and nanoparticles [7]. On the other hand, the surface plasmon localization (SPL) technique on metallic nanoparticles is used as one of the most efficient methods used to improve the optical absorption of organic solar cells [8]. Much effort has been made in the literature using different techniques to increase the PCE of a solar cell using plasmonic nanoparticles. Williamson A. et al. introduced a theoretical study of a periodic plasmonic solar cell to enhance the PCE [9]. A novel design for high efficiency was produced by reducing the thickness of the active layer of the solar cell, which led to enhancing the performance through the superposition of tooth-grating structures. AuNPs are used to enhance the absorption of the solar cell, which decorate the ZnO nanowires, in the visible and infrared regions [10,11,12,13,14,15]. AuNPs distributed on the ZnO nanowires and ZnO layer are used as a highly sensitive biosensor and high-efficient solar cells [16,17,18,19,20,21,22]. The NPs’ shapes and materials are the main parameter that affect the solar cell and sensor performance [22,23,24,25]. The authors used the FDTD method to optimize the overall spectral response of the cell.

Kao C. et al. used gold nanoparticles (AuNPs) in inverted organic photovoltaic devices to enhance the solar cell performance [26]. The photocurrent and fill factors were improved after using gold nanoparticles in the solar cell nanostructure. The authors conclude that the rough surfaces might increase the device and consequently decrease the power conversion efficiency [26].

Improved optical absorption was introduced by Liu F. et al. by distributing silver nanoparticles (AgNPs) on the interface layer between (PEDOT:PSS) poly (3,4-ethylenedioxythiophene):poly(styrenesulfonate) and (P3HT:PCBM) poly(3-hexylthiophene):(6,6)-phenyl-C61-butyric-acid -methyl ester layers [8].

On the other hand, the absorption reached almost 100% when the silver nanoparticles were distributed, as introduced by Gasparini N. et al. [27]. The obtained power conversion efficiency from the introduced organic solar cell with a thick active layer was 11%, theoretically.

A nanohole photoactive layer was used to enhance the overall efficiency of a polymer solar cell to almost 6.7% [28]. The proposed structure was compared with another model introduced by Tumbleston J. et al. [29] where a photonic crystal photoactive layer was used to reach 5.03% improvement in the power conversion efficiency.

Jiang X. and et al. improved the electrical conductivity of PEDOT:PSS to 35% by adding a graphene layer to the organic solar cell [30]. The carrier mobility and overall efficiency improved by adding charge transport pathways in the hole transport layer. In the previous work [7], and by using a graphene layer and AuNPs distributed on the top of the solar cell, the overall efficiency was improved to 8.94% and the short circuit current density *J_sc_* = 17.32 mA/cm^2^.

The main objective of this work was to obtain a high efficiency polymer solar cell by increasing the short circuit current density and the light absorption in the P3HT:PCBM active layer. The results were obtained by using a finite difference time domain method, where the Lumerical FDTD solutions software package was used. The optimization of the periodic grating nanostructure of the ZnO layer and PEDOT:PSS layer was determined by simulating the proposed structure. Next, the optimum radius of the distributed AuNPs in the active layer was also considered in this paper. In addition, the effect of temperature on the short circuit current density and the overall efficiency of the solar cell were also calculated. Furthermore, the optical absorption from the proposed structure at different polarization angle was also introduced. Finally, the power, electric field, and magnetic field distribution inside the polymer solar cell are illustrated in this paper.

## 2. Mathematical Model

The most common model to calculate the overall organic solar cell efficiency is the single diode model [28], where the short circuit current density, *J_sc_*, can be calculated using Equation (1) by assuming that an incident photon will produce an electron [7].

(1) $$J_{sc}=\frac{e}{hc}\int I\left(\lambda \right)A\left(\lambda \right)\lambda \;d\lambda$$

where *h* is the Planck’s constant; *c* is the speed of light; *e* is the electron charge; and *I(λ)*, *A(λ)* are the standard air mass 1.5 (AM1.5) spectral irradiance and the optical absorption, respectively. On the other hand, the open-circuit voltage, *V_oc_*, of P3HT:PCBM is assumed to be a fixed value equal to 0.62 V as it is wholly dependent on the energy level of the used material [28]. Hence, the fill factor, *FF*, can be calculated using Equation (2).

(2) $$FF=\frac{\frac{V_{oc}}{V_{t}}-ln\left(\frac{V_{oc}}{V_{t}}+0.72\right)}{\frac{V_{oc}}{V_{t}}+1}$$

where *V_t_* is the thermal voltage equal to 0.025 at room temperature. The maximum output power from the solar cell, *P_max_*, is calculated using Equation (3).

(3) $$P_{max}=0.62\times J_{sc}\times FF\;$$

Hence, the overall solar cell efficiency, *η*, can be calculated as a ratio of maximum output power to solar input power.

On the other hand, AuNPs distributed on the substrate layer will change the absorbed optical power in the active layer and depend on the maximum reflectivity value. Nanoparticle shape has a significant effect on the transmitted optical power as well as the relative permittivity of the gold nanoparticles and the dielectric function of the surrounding medium [7]. The maximum value of the absorbed power is located at *λ_max_*, which can be calculated using Equation (4).

(4) $$\lambda_{max}=\frac{M}{g}{\left(\frac{\varepsilon_{Au}\varepsilon_{m}\left(\lambda_{max}\right)}{\varepsilon_{m}+\varepsilon_{Au}\left(\lambda_{max}\right)}\right)}^{1/2}$$

where *ε_m_* is the permittivity of the surrounding medium; *ε_Au_* is a gold nanoparticles dielectric constant at corresponding *λ_max_*; *g* is an integer and depends on the surrounding material and gold nanoparticle shape, which is calculated by the simulation tool; and *M* is structural periodicity, which simulated in this work for different values. Hence, the dielectric permittivity can be expressed by using a multi-oscillator Drude–Lorentz model [7] as given in Equation (5):

(5) $$\varepsilon_{Au}=\varepsilon_{\infty }-\frac{\omega_{D}^{2}}{\omega^{2}+j\omega \gamma_{D}}-\overset{6}{\underset{k=1}{\sum }}\frac{\delta_{k}\omega_{k}^{2}}{\omega^{2}-\omega_{k}^{2}+2j\omega \gamma_{k}}$$

where *ε_ꝏ_* is the gold high-frequency dielectric permittivity at; *ω_D_* and *γ_D_* are the plasma and collision frequencies of the free electron gas; *δ_k_* is the amplitude of the Lorentz oscillator; *ω_k_* is the resonance angular frequencies; and *γ_k_* is the damping constants for *k* value from 1 to 6.

## 3. Proposed Solar Cell Structure

**Figure 1:**
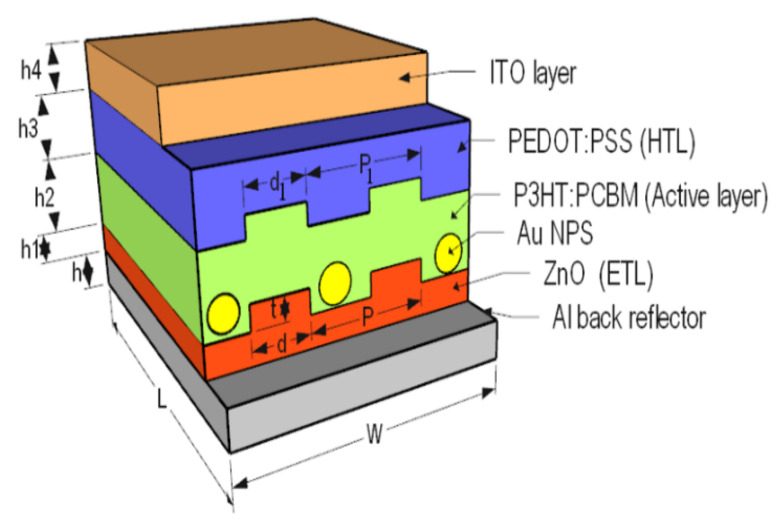
Schematic diagram of the proposed structure.

The proposed structure was designed and analyzed using an electromagnetic wave solver, Lumerical FDTD solutions software, which used the designed polymer solar cell’s finite difference time domain method. In this simulation analysis, unit cell dimensions were simulated and assumed to be *W* = 460 nm (width) and *L* = 800 nm (length) with aluminum, *Al*, as a back contactor of thickness *h* = 300 nm, as it was used as a cathode. The electron transport layer is the ZnO material grown above the *Al* layer with thickness *h*1 = 60 nm with periodic grating height *t* = 35 nm, the grating width, *d*, and grating period of ZnO layer, *P*, will be optimized, as illustrated in Figure 1. The height of the active layer, P3HT:PCBM, was *h*2 = 200 nm, which was placed on the top of the ZnO layer, where the PEDOT:PSS layer was used as a hole transport layer (HTL) with thickness *h*3 = 50 nm. ITO material covered the structure with height *h*4 = 170 nm and plasmonic NPs were distributed in the active layer with a distance between each NP *Y* = 150 nm in the y-direction.

A one-unit cell was simulated, and the boundary conditions were considered as the periodic structure in both *x*- and *y*-directions and perfect matching layer (PML) in the *z*-direction. The used light source is a plane wave with a wavelength band 400–700 nm and offset time of 7.5 fs. In addition, the solar generation calculation region was placed in the active region to calculate the short circuit current density.

## 4. Results and Discussion

**Figure 2:**
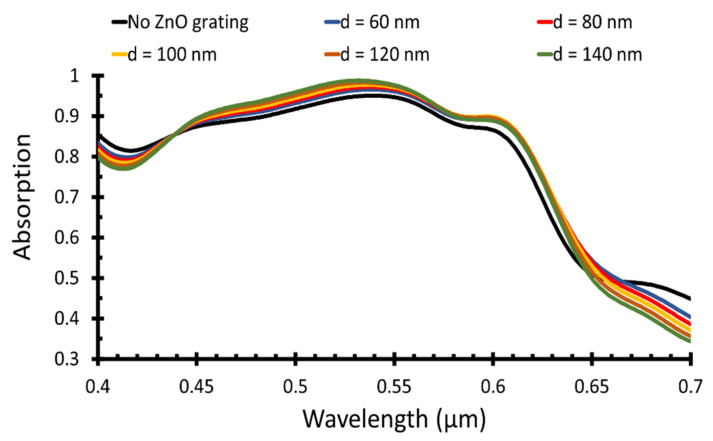
Absorption of different ZnO grating periods versus optical wavelength.

The main objective was to maximize the absorbed power in the active layer, which led to increase the short circuit current density and consequently the power conversion efficiency of the solar cell. Figure 2 illustrates the light absorbed in the active region of the polymer solar cell at different ZnO grating width, *d* = 50 nm to 150 nm with a step of 10 nm, and compared to the absorption when there was no ZnO grating. Selected values of ZnO grating width are illustrated in Figure 2. As shown in Figure 2, there was no major difference in the light absorption for different grating widths. However, the *J_sc_* showed slight changes in the values, which led to different power conversion efficiency values, as given in Table 1.

In Table 1, the short circuit current density and the PCE are given at different ZnO grating widths, giving the height *J_sc_* =14.47 mA/cm^2^ and *η* = 7.56% at grating width *d* = 100 nm.

**Figure 3:**
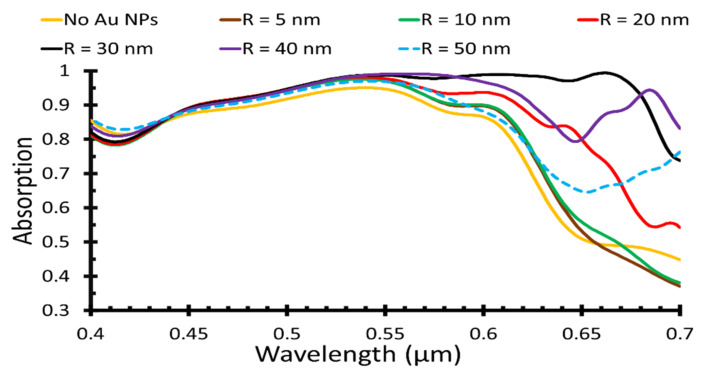
The light absorption of different AuNP radii with the wavelength.

AuNPs were then distributed between the ZnO gratings, as shown in Figure 1, with different radii. Figure 3 shows the light absorption of different AuNP radii, *R* = 5, 10, 20, 30, 40, and 50 nm, which was compared to the absorptance when AuNPs were not distributed. The effect of surface plasmon resonance shifted the maximum wavelength to almost 650 nm when P3HT was used as an active layer, as illustrated in Figure 3, which was clearly observed at higher AuNP radii, *R* = 30, 40, and 50 nm. 

As clearly shown in Figure 3, the maximum absorption wavelength increased when the AuNP radius increased, so the maximum absorption can be acquired at NP radius *R* = 30 nm, at wavelengths from 0.6 nm to 0.68 nm. However, the maximum short circuit current density was 15.93 mA/cm^2^, and an overall efficiency of 8.33% could be achieved at NP radius *R* = 40 nm as they were calculated over the visible band region, as given in Table 2. For *R* = 50 nm, the absorption was enhanced and shifted to a higher wavelength in the near infrared region, so the short circuit current density and PCE measured in the visible region had lower values than the other radii.

**Figure 4:**
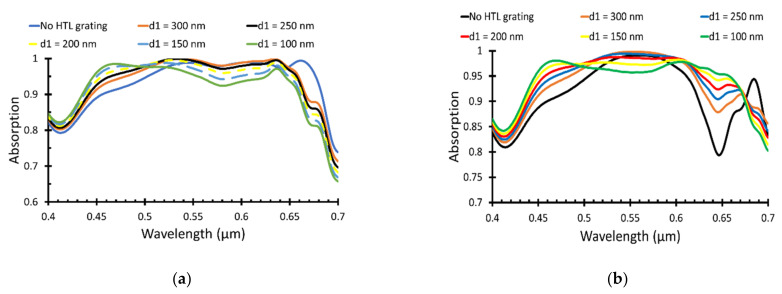
Light absorption (**a**) at NP radius of 30 nm and (**b**) at NP radius of 40 nm.

Therefore, both values were considered, and the HTL grating width was simulated for both radii of 30 and 40 nm. Figure 4a,b shows the absorption versus wavelength for different HTL grating width sat the Au NP radii of 30 nm and 40 nm, respectively.

The absorptance attained from HTL grating width, *d*1, 100 nm at NP radius *R* = 40 nm was the maximum value as shown in Figure 4b, and the short circuit current density was 18.1 mA/cm^2^, which gives a PCE equal to 9.46%, as illustrated in Table 3.

**Figure 5:**
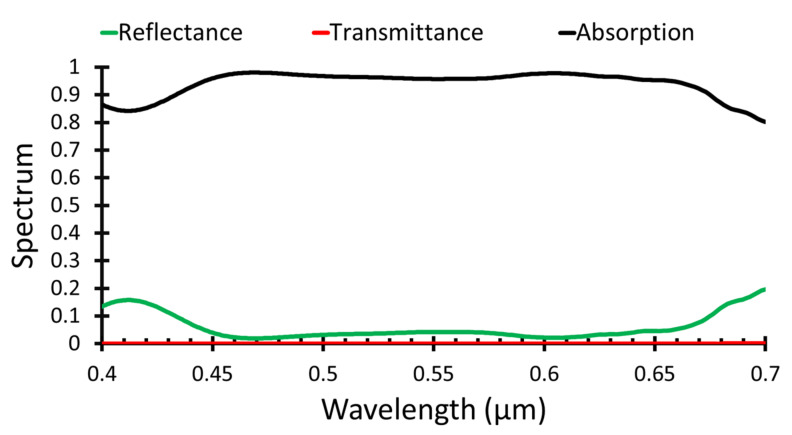
Absorbance, transmittance, and reflectance spectra of the proposed structure.

The overall absorbance, transmittance, and reflectance spectra of the obtained maximum performance is shown in Figure 5. The optimum ZnO grating width was *d* = 100 nm, the AuNP radius was 40 nm, and the HTL grating width was 100 nm.

**Figure 6:**
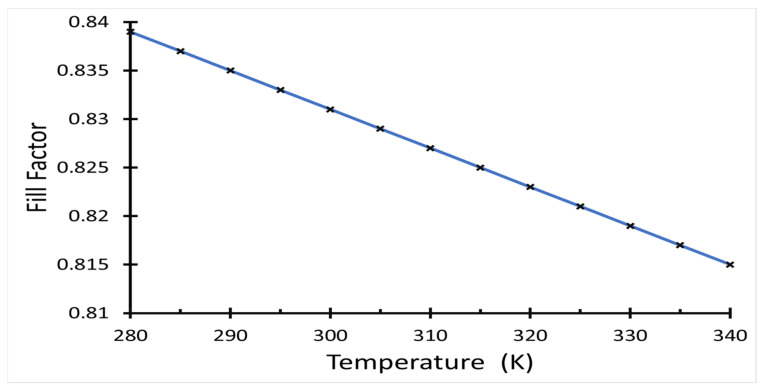
Fill factor of the solar cell as a function of temperature.

Using Equation (2), the effect on temperature on the fill factor of the solar cell can be calculated as given in Figure 6, where the maximum *FF* value was found at temperature T = 280 K for a normal temperature range from 280 K to 340 K.

**Figure 7:**
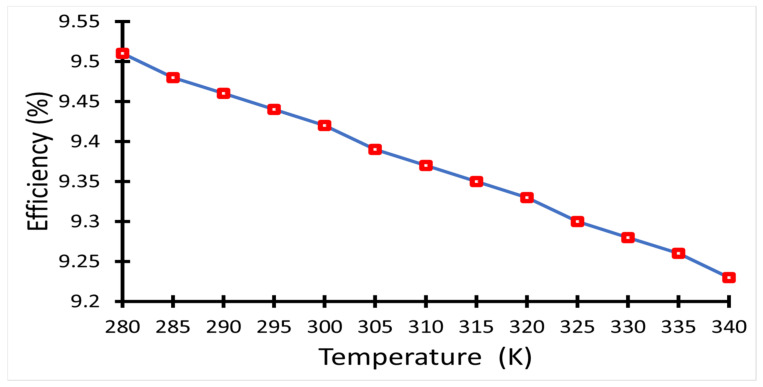
PCE of the solar cell as a function of temperature.

The PCE values were then calculated for the same temperature range as given in Figure 7, where the efficiency decreased as the temperature increased. The effect of temperature on the solar cell fill factor was calculated using Equation (2), then the maximum output power was determined using Equation (3). Hence, the PCE as a function of temperature can be obtained, which is directly proportional with maximum output power and consequently with the fill factor. The maximum efficiency 9.51% was obtained at T = 280 K, and the minimum PCE was 9.24% at T = 340 K.

**Figure 8:**
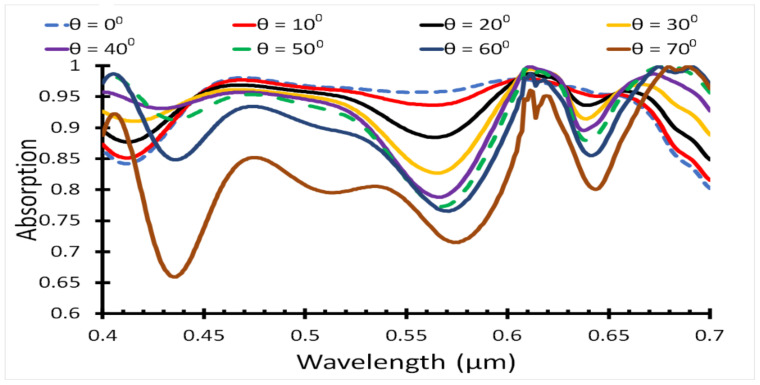
Absorbed light in the active region for different incident angle.

The polarization of the incident light plays an important role in the solar cell efficiency, so the effect of polarization on the solar cell was also simulated, as illustrated in Figure 8.

The absorption of the incident light was simulated for different incident angles, starting from 0° to 70° with a step of 10°, as shown in Figure 8. Table 4 shows the short circuit current density and PCE for different incident angles, where the *J_sc_* and PCE values decreased with an increase in the incident angle. The absorption was almost flat in the mid-band, λ = 0.45 nm to 0.65 nm, for direct incident light, *θ* = 0°. Moreover, increasing polarization led to decreasing the absorption in the mid-band and increasing the absorption in the outer-bands lower than 0.45 nm and higher than 0.65 nm.

The obtained open-circuit voltage, fill factor, short circuit current density, and overall efficiency from the proposed nanostructure were then compared to other nanostructures. The conventional PC structure [29], NH structure [28], ZnO pillars and plasmonic NP structure [7], and O-GNP structure [6] were compared to the proposed structure as illustrated in Table 5. The open-circuit voltage and fill factor values were equal for all introduced structures of 0.62 V and 0.83, respectively, except for the O-GNP structure, which were *V_oc_* = 0.26 V and *FF* = 0.69. The maximum short circuit current density is given by the O-GNP structure, *J_sc_* = 44.32 mA/cm^2^. However, the obtained PCE was 7.84%. On the other hand, the proposed structure obtained *J_sc_* = 18.11 mA/cm^2^ and *η* = 9.46.

**Figure 9:**
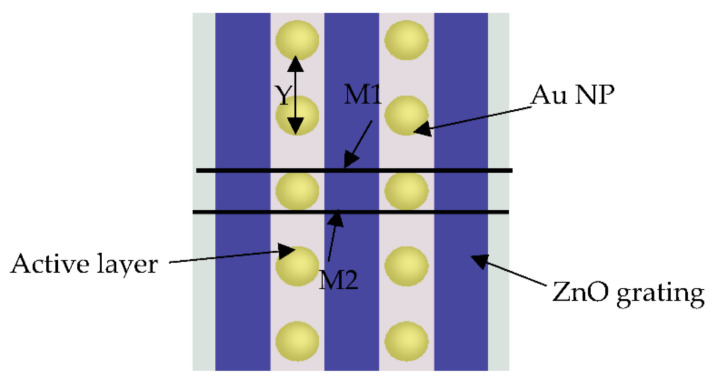
Top view of the proposed structure illustrates the location of monitors *M*1 and *M*2.

Optical power, electric and magnetic fields distribution are represented at two different vertical monitors: the first monitor, *M*1, passes through ELT, the active layer, HTL and AuNPs, however, the second monitor, *M*2, passes through ELT, the active layer, and HTL but does not pass through AuNPs, as given in Figure 9.

**Figure 10:**
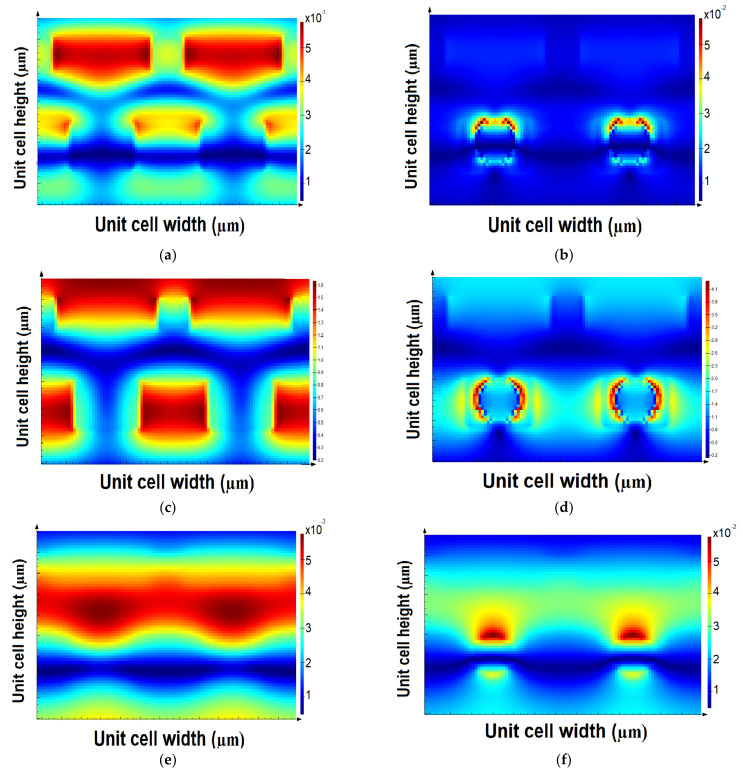
Optical power distribution on vertical planes (**a**) for the first monitor, *M*1 (**b**) for the second monitor, *M*2, electric field distribution (**c**) for the first monitor, *M*1 (**d**) for the second monitor, *M*2, and magnetic field distribution (**e**) for the first monitor, *M1* and (**f**) for the second monitor, *M*2.

The distribution of optical power illustrates that the HTL gratings absorbed the incident optical power and retransmitted it to the active layer before being collected by ZnO gratings, as shown in Figure 10a. On the other hand, more optical power was absorbed by the proposed structure in the HTL gratings and retransmitted to the active layer, which received more power by the AuNPs before retransmitting the power to the ETL, as illustrated in Figure 10b.

Based on the same concept, the electric field was confined HTL and ETL gratings in the case of not AuNPs, and a high electric field was absorbed by the AuNPs if they were distributed on the ZnO layer, as illustrated in Figure 10c,d. In Figure 10c, the light was absorbed in the HTL and ETL gratings, which is illustrated in dark red, however, in Figure 10b, AuNPs absorbed higher optical power, which is shown in red with respect to the light blue of the gratings’ optical absorption. However, the magnetic field distribution on both monitors *M*1 and *M*2 are shown in Figure 10e,f, where a high magnetic field was absorbed in the HTL gratings before transmitting it to the ETL gratings through the active layer and AuNPs.

## 5. Conclusions

In this paper, optical absorption, open-circuit voltage, short circuit current density, fill factor, and power conversion efficiency of a polymer solar cell were simulated using the FDTD method. A high improvement in the electrical and optical properties in the active layer was accomplished by using the HLT grating period of 100 nm and ETL grating period of 100 nm. The distribution of AuNPs enhanced the light absorption, where the optimum Au NP radius was 40 nm, which produced a short circuit current density *J_sc_* = 18.11 mA/cm^2^ and PCE = 9.46%. In addition, the effect of temperature on the performance of the fill factor. The solar cell’s overall efficiency was also provided for a temperature range from 280 K to 340 K. The maximum performance of the organic solar cells was obtained at a temperature of 208 K, where the fill factor was 0.839. The power conversion efficiency was 9.5%. Finally, the optical power distribution, electric and magnetic fields, with and without AuNPs, were simulated and analyzed through the given nanostructure. Hence, the ETL gratings absorbed the optical power and retransmitted to the active layer to generate an electron hole pair before separating them and generating the electric current.

## Figures and Tables

**Table 1:** Short circuit current density and overall efficiency for different ZnO grating widths.

ZnO Gratings, *d* (nm)	*J_sc_* (mA/cm^2^)	Efficiency (%)
Flat	13.50	7.05
50	14.05	7.34
60	13.98	7.30
70	14.31	7.47
80	14.21	7.43
90	14.17	7.40
100	14.47	7.56
110	14.33	7.49
120	14.37	7.51
130	14.65	7.66
140	14.54	7.55
150	14.36	7.50

**Table 2:** *J_sc_* and PCE for different AuNP radii.

AuNP Radius (nm)	*J_sc_* (mA/cm^2^)	Efficiency (%)
No AuNPs	13.50	7.05
5	15.16	7.92
10	15.42	8.02
20	15.55	8.12
30	15.44	8.07
40	15.93	8.33
50	13.00	6.79

**Table 3:** *J_sc_* and PCE at AuNP radii of 30 nm and 40 nm.

HTL Gratings, *d*1 (nm)	*J_sc_* (mA/cm^2^)	Efficiency (%)	*J_sc_* (mA/cm^2^)	Efficiency (%)
	*R* = 30 nm		*R* = 40 nm	
Flat	15.44	8.07	15.93	8.32
300	16.41	8.57	16.71	8.73
250	16.93	8.85	17.28	9.03
200	17.04	8.9	17.45	9.12
150	17.53	9.16	17.97	9.39
100	17.44	9.11	18.11	9.46

**Table 4:** *J_sc_* and PCE for different light incident angles.

Light Incident Angle	*J_sc_* (mA/cm^2^)	Efficiency (%)
0°	18.11	9.46
10°	17.98	9.39
20°	17.82	9.31
30°	17.39	9.08
40°	16.66	8.71
50°	15.73	8.22
60°	14.97	7.82
70°	15.19	7.94

**Table 5:** *V_oc_*, *FF*, *J_sc_*, and PCE for different related nanostructures compared to the proposed structure.

Structure	*V_oc_* (V)	*FF*	*J_sc_* (mA/cm^2^)	Efficiency (%)
Conventional PC [29]	0.62	0.83	10.13	5.03
NH [28]	0.62	0.83	13	6.71
ZnO pillars and plasmonic NPs [7]	0.62	0.83	17.32	8.94
O-GNPs [6]	0.26	0.69	44.32	7.84
Proposed structure	0.62	0.83	18.11	9.46

## Data Availability

Not applicable.
